# Increased Thymic B Cells but Maintenance of Thymic Structure, T Cell Differentiation and Negative Selection in Lymphotoxin-α and TNF Gene-Targeted Mice

**DOI:** 10.1155/2000/13492

**Published:** 2000

**Authors:** Adrian P. Grech, D. Sean Riminton, Melinda J. Gabor, Charles L. Hardy, Jonathon D. Sedgwick, Dale I. Godfrey

**Affiliations:** 1 Centenary Institute of Cancer Medicine and Cell Biology Locked bag No. 6 Newtown NSW 2042 Australia; 2 Department of Immunology St Jude Children's Research Hospital 332 North Lauderdale PO Box 318 Memphis TN 38101-0318 USA; 3 DNAX Research Institute 901 California Avenue Palo Alto CA. 94301 USA; 4 Department of Pathology and Immunology Monash Medical School Commercial Road Prahran VIC. 3181 Australia

**Keywords:** Thymus, Tumor Necrosis Factor, Lymphotoxin, T lymphocytes, transgenic/knockout

## Abstract

TNF, lymphotoxin (LT) and their receptors are expressed constitutively in the thymus. It remains unclear whether these cytokines play a role in normal thymic structure or function. We have investigated thymocyte differentiation, selection and thymic organogenesis in gene targeted mice lacking LTα, TNF, or both (TNF/LTα^-/-^). The thymus was normal in TNF/LTα^-/-^ mice with regard to cell yields and stromal architecture. Detailed analysis of αβ and γδ T cell-lineage thymocyte subsets revealed no abnormalities, implying that neither TNF nor LT play an essential role in T cell differentiation or positive selection. The number and distribution of thymic CD11c^+^ dendritic cells was also normal in the absence of both TNF and LTα. A three-fold increase in B cell numbers was observed consistently in the TNF/LTα^-/-^ thymus. This phenotype was due entirely to the LTα deficiency and associated with changes in the hemopoietic compartment, rather than the thymic stromal compartment of LTα^-/-^ mice. Finally, specific Vβ8^+^ T cell deletion within the thymus following intrathymic injection of staphylococcal enterotoxin B (SEB) was TNF/LT independent. Thus, despite the presence of these cytokines and their receptors in the normal thymus, there appears no essential role for either TNF or LT in development of organ structure or for those processes associated with T cell repertoire selection.


*Developmental Immunology*, 2000, Vol. 8(1), pp. 61-74
Reprints available directly from the publisher
Photocopying permitted by license only

© 2000 OPA (Overseas Publishers Association)
N.V. Published by license under
the Harwood Academic Publishers imprint,
part of The Gordon and Breach Publishing Group.
Printed in Malaysia

### Increased Thymic B Cells but Maintenance of Thymic Structure, T Cell Differentiation and Negative Selection in Lymphotoxin-α and TNF Gene-Targeted Mice

ADRIAN P. GRECH[a], D. SEAN RIMINTON[a], MELINDA J. GABOR[a], CHARLES L. HARDY[b], JONATHON D. SEDGWICK[c*] and DALE I. GODFREY[d†]

[a]*Centenary Institute of Cancer Medicine and Cell Biology, Locked bag No. 6, Newtown 2042, NSW, Australia,* [b]*Department of Immunology, St Jude Children's Research Hospital, 332 North Lauderdale, PO Box 318, Memphis, TN 38101–0318, USA,* [c]*DNAX Research Institute, 901 California Avenue, Palo Alto, CA. 94301, USA and* [d]*Department of Pathology and Immunology, Monash Medical School, Commercial Road, Prahran, VIC. 3181, Australia*

TNF, lymphotoxin (LT) and their receptors are expressed constitutively in the thymus. It remains unclear whether these cytokines play a role in normal thymic structure or function. We have investigated thymocyte differentiation, selection and thymic organogenesis in gene targeted mice lacking LTα, TNF, or both (TNF/LTα$^{-/-}$). The thymus was normal in TNF/LTα$^{-/-}$ mice with regard to cell yields and stromal architecture. Detailed analysis of αβ and γδ T cell-lineage thymocyte subsets revealed no abnormalities, implying that neither TNF nor LT play an essential role in T cell differentiation or positive selection. The number and distribution of thymic CD11c$^{+}$ dendritic cells was also normal in the absence of both TNF and LTα. A three-fold increase in B cell numbers was observed consistently in the TNF/LTα$^{-/-}$ thymus. This phenotype was due entirely to the LTα deficiency and associated with changes in the hemopoietic compartment, rather than the thymic stromal compartment of LTα$^{-/-}$ mice. Finally, specific Vβ8$^{+}$ T cell deletion within the thymus following intrathymic injection of staphylococcal enterotoxin B (SEB) was TNF/LT independent. Thus, despite the presence of these cytokines and their receptors in the normal thymus, there appears no essential role for either TNF or LT in development of organ structure or for those processes associated with T cell repertoire selection.

*Keywords:* Thymus, Tumor Necrosis Factor, Lymphotoxin, T lymphocytes, transgenic/knockout

### INTRODUCTION

The thymus provides the microenvironment necessary for the development of T cells from lymphoid progenitor cells as well as for selective elimination of T cells that are potentially autoreactive. This process, termed negative selection, inhibits maturation of those cells expressing receptors reactive to self antigens and hence promotes the maintenance of self tolerance (Kisielow and von Boehmer, 1995). Intrathymic negative selection involves active elimination of cells through the induction of apoptosis

* Correspondence: Dale I. Godfrey, Department of Pathology and Immunology, Monash Medical School, Commercial Road, Prahran, VIC. 3181, Australia. Phone: 61 3 99030761, Fax: 61 3 99030731, email: Dale.Godfrey@med.monash.edu.au
† Jonathon D. Sedgwick, DNAX Research Institute, 901 California Avenue, Palo Alto, CA. 94301, USA. Phone: 4158529196. Fax: 4151961200. e-mail: jon.sedgwick@dnax.org

(programmed cell death) in negatively selected cells (Surh and Sprent, 1994). This appears to involve a signal through the TCR of the developing thymocyte in conjunction with second signal(s) which are as yet poorly defined (Page et al., 1993; Page et al., 1996).

Both TNF and LT have a well documented role in the induction of apoptosis in general, inducing programmed cell death through signalling via the p55 TNF receptor (TNFRI) and LTβR (Sarin et al., 1995; Ware et al., 1995; Browning et al., 1996; Nagata, 1997). Several studies have demonstrated constitutive expression of TNF in the thymus *in vivo* (Giroir et al., 1992; Murphy et al., 1992; Wolf and Cohen, 1992; Deman et al., 1996), moreover, the thymus was the only organ in which the TNF promoter was constitutively active (Giroir et al., 1992). Similarly, LTα and LTβ are constitutively expressed in the thymus (Wolf and Cohen, 1992; Pokholok et al., 1995), as are the TNF receptors TNFRI (p55) and TNFRII (p75) to which TNF and the LTα3 homotrimers bind (Ryffel et al., 1991; Tartaglia et al., 1991; Murphy et al., 1994). The receptor for the membrane bound LTα/β complex, LTβR, has been shown to be highly expressed in thymic epithelial tissue (Ware et al., 1995). TNF is produced by both immature and mature thymocyte subsets following stimulation *in vitro* (Fischer et al., 1991; Zlotnik et al., 1992). Several studies have implicated a role for TNF in T cell differentiation and/or proliferation, at least *in vitro*. TNF has been implicated as an important stimulatory factor at several points of T cell differentiation (Suda et al., 1990; Suda et al., 1990; Suda and Zlotnik, 1992; Suda and Zlotnik, 1992; Zuniga-Pflucker et al., 1995). Conversely, TNF leads to rapid apoptosis of immature thymocytes in cell suspension (Hernandez-Caselles and Stutman, 1993) and mice overexpressing TNF in their T cells have reduced $CD4^+CD8^+$ double positive (DP) thymocyte numbers (Probert et al., 1993). TNF expression also coincides with the ability of fibroblast antigen presenting cells to induce negative selection *in vitro* (Page et al., 1993). Although many biological effects of TNF appear to occur via TNFRI, TNFRII appears to be both necessary and sufficient for at least some aspects of TNF mediated thymocyte stimulation (Tartaglia et al., 1991; Grell et al., 1998). LTα/β and TNF play an essential role as mediators of lymphoid organ ontogeny and maintenance of lymphoid structure (De Togni et al., 1994; Pasparakis et al., 1996; Koni et al., 1997; Korner et al., 1997; Cook et al., 1998), at least in part through induction of B and T cell homing chemokines (Ngo et al., 1999).

Despite this abundance of indirect evidence suggesting a role for TNF and LT in thymic T cell development, clear support for such a role *in vivo* has been elusive. Antibody blocking experiments (Sytwu et al., 1996) have failed to indicate a role for TNF in T cell development or negative selection *in vivo*. However, such studies may be complicated by the difficulty of completely saturating antigenic sites in the thymus, particularly with non-saturating doses of antibody (Gabor et al., 1997), due to the, at least partially effective, blood thymus barrier (Raviola and Karnovsky, 1972). Studies with TNFRI/RII deficient mice (Pfeffer et al., 1993; Page et al., 1998), or transgenic mice expressing soluble LTβR and TNFRI (Ettinger et al., 1998), showed no major thymic abnormalities although *in vitro*, but not *in vivo* negative selection, was impaired in the absence of TNFR signalling (Page et al., 1998). Given the constitutive expression of TNF/LT molecules and their receptors in the thymus, it is surprising that no clear role for these factors in thymus/T cell development has been identified thus far. However, considering that TNF and LT share some functional characteristics, it remained possible that redundancy in the activity of these factors, and/or their receptors, had obscured their role in thymus/T cell development in these studies. Specifically, signalling of membrane LTα/β via the LTβR would be maintained in TNFRI/RII deficient mice (Page et al., 1998), while signalling via TNFRII may be maintained in TNFRI deficient mice (Pfeffer et al., 1993) and transgenic mice expressing soluble LTβR and TNFRI (Ettinger et al., 1998).

We hypothesized that TNF and/or LT, through effects on lymphoid ontogeny or through their capacity to induce apoptosis, may have an as yet unidentified function in thymic physiology/T cell development. To test this, we used previously described C57BL/6 mice with targeted disruption of the TNF, TNF/LTα (Korner et al., 1997) or LTα

(Riminton et al., 1998) genes. Mice in which the gene for LTα is deleted lack both the secreted LTα3 and predominant membrane LTα1β2 forms of the LT molecule, the latter by virtue of the fact that expression of the LTβ molecule at the cell-surface fails or is non-functional in the absence of LTα (Browning et al., 1993). Thus, TNF/LTα$^{-/-}$ mice are completely LT and TNF-deficient, and not only lack lymph nodes and Peyer's patches (Eugster et al., 1996) but exhibit profound changes to the microarchitecture of the spleen that are greater in magnitude to that seen in mice that lack either cytokine alone ((Korner et al., 1997; Riminton et al., 1998) and unpublished observations). We have used these mice to perform a comprehensive analysis of thymocyte subsets and thymic structure. This included detailed examination of thymocyte differentiation, including positive and negative selection (mediated by the superantigen SEB), as well as non-T lineage cells (B cells, macrophages, dendritic cells and thymic stromal cells). The results indicated no major differences in T lineage development relative to wild-type (WT) mice. However, increased numbers of B cells were identified in the TNF/LTα$^{-/-}$ thymus by flow cytometry and immunohistology and this was attributed to the absence of LT. In summary, this study indicates that TNF and LT at best play a dispensable or redundant role in most aspects of intrathymic T cell development.

### RESULTS

### Thymic Structure is Maintained in the Absence of TNF and LT

To determine whether the absence of both TNF and LT affected the integrity of the thymus, TNF/LTα$^{-/-}$ thymuses were examined for gross structural abnormalities and for microarchitectural changes. These thymuses were macroscopically normal, comparable to WT thymuses in terms of tissue mass and cellularity. However, TNF/LTα$^{-/-}$ thymocyte viability, as assessed by Trypan blue dye exclusion, was slightly but reproducibly lower than that of WT mice (Table I).

TABLE I Thymic mass and cellularity in TNF/LTα$^{-/-}$ mice

| *Variable* | *WT*[a] | *TNF/LTα*$^{-/-a}$ |
|---|---|---|
| Thymic mass (mg) | 69±2[b] (17)[c] | 65±5 (17) |
| Total thymocytes (x10$^{-8}$) | 2.1±0.1 (21) | 2.0±0.1 (21) |
| Total viable thymocytes (x10$^{-8}$)[d] | 1.9±0.1 (21) | 1.7±0.1 (21) |
| Non-viable thymocytes (%) | 8.7±0.6[e] (21) | 11.8±0.8[e] (21) |

Thymuses were removed from 6–10 week old female WT and TNF/LTα$^{-/-}$ mice, weighed then disrupted to release thymocytes which were counted and total or viable yields determined. Within this age range, no age-related differences were observed for any parameter examined.

a. Values are per thymus
b. Standard error of the mean
c. Number of thymuses examined
d. Viability determined by trypan blue exclusion
e. Significantly different (Mann-Whitney U test, $p \leq 0.01$)

Thymic stromal compartments were examined by immunohistology with mAb that facilitated the identification of particular regions of the thymus. These included molecules such as MHC class II, expressed by the fine cortical epithelial network and more densely expressed in the thymic medulla, and MTS33, expressed by cortical thymocytes and isolated medullary epithelial cell clusters (Godfrey et al., 1990) both of which enabled clear distinction between cortex and medulla (Figure 1). No differences were detected in the thymus from WT versus TNF/LTα$^{-/-}$ mice. Lymphocyte markers CD4 (not shown) and CD8 (Figure 1), both densely label the cortex and are less frequent in the thymic medulla. These also revealed no differences between the TNF/LTα$^{-/-}$ and WT thymus. MTS12 (not shown) and MTS16 (Figure 1) label thymic blood vessels and associated connective tissue lining the perivascular space, respectively (Godfrey et al., 1990). Again, the staining pattern with both mAb was identical in thymi from WT and TNF/LTα$^{-/-}$ mice illustrating conserved cortical/medullary compartmentalisation and normal vascular integrity. Thymic dendritic cells, identified by CD11c expression (Figure 1), were confined to the thymic medulla in both WT and TNF/LTα$^{-/-}$ mice.

### Major Thymocyte Subsets are Maintained in the TNF/LTα$^{-/-}$ Thymus

To determine whether the absence of TNF and LTα affected proportions of thymocyte subsets, multi-color flow cytometric analysis of isolated cells was performed (Figure 2). Plots of CD4 vs CD8 thymocytes from WT and TNF/LTα$^{-/-}$ mice showed no difference in the proportions of CD4$^-$CD8$^-$, CD4$^+$CD8$^+$, CD4$^+$CD8$^-$and CD4$^-$CD8$^+$ populations. αβTCR (Figure 2) and γδTCR expression (not shown) of each of these subsets was also examined revealing marginally fewer αβTCR$^+$cells within the CD4$^-$CD8$^-$ population in the TNF/LTα$^{-/-}$ thymus ($p<0.05$, Mann-Whitney U-test). This was not reflected in a loss of NK1.1$^+$αβTCR$^+$ cells (NKT cells) that represent a significant proportion of αβTCR$^+$CD4$^-$CD8$^-$ thymocytes (Levitsky et al., 1991), as these were present in normal numbers in TNF/LTα$^{-/-}$ thymuses (not shown). HSA expression was also tested as this molecule is down-regulated at a late stage in medullary thymocyte development, well after thymocytes reach the CD4 or CD8 single positive (SP) stage. Again, no differences were detected between WT and TNF/LTα$^{-/-}$ mice (not shown).

### Proportions of Non-T-Lineage Cells in the Thymus of TNF/LTα$^{-/-}$ Mice

The expression of a range of markers defining non-T lineage cells within the heterogeneous CD4$^-$CD8$^-$ thymocyte subpopulation was examined. Equivalent numbers of CD11c$^+$ cells (highly enriched for dendritic cells) were found in both WT and TNF/LTα$^{-/-}$ mice, consistent with the data of Figure 1 (not shown). In contrast, a three-fold increase in B220$^+$ cells and a 2-fold increase in Mac-1$^+$ cells was detected in CD4-CD8- thymocytes from TNF/LTα$^{-/-}$ compared to WT mice (Figure 2).

### Most B220$^+$ Cells in the TNF/LTα$^{-/-}$ Thymus Exhibit a Peripheral B2 Phenotype

The majority (>95%) of mature B cells found in the blood and lymphoid tissues of mice are of the B2 type with a well defined phenotype (Hardy and Hayakawa, 1994). Most (~90%) B220$^+$ cells in the spleen are IgD$^{high}$ while few express the CD5 antigen that is characteristic of B1 cells, found particularly in the peritoneal cavity (Kipps, 1989). In contrast, a high proportion of B cells in the thymus are CD5$^+$Mac-1$^+$ (Miyama-Inaba et al., 1988). These B cells are thought to develop within the thymus from a local progenitor population (Mori et al., 1997). A more detailed phenotypic analysis was performed to determine whether the increase in B220$^+$cells within the CD4$^-$ CD8$^-$ thymic population in TNF/LTα$^{-/-}$ mice represented a selective expansion of thymic B cells (Figure 3A). Of B220$^+$ cells in the WT mouse, around 60% were CD5$^{+ve}$ and around 65% IgD$^{-ve}$, confirming the over-representation of this atypical subset in the thymus. In contrast, of B220$^+$cells in the TNF/LTα$^{-/-}$, 25% were CD5$^{+ve}$ and around 50% IgD$^{-ve}$. Thus, B cells in the TNF/LTα$^{-/-}$ thymus were enriched for normal peripheral type B2 cells rather than the thymic variety. This also suggested that the more numerous Mac-1$^+$ cells in the TNF/LTα$^{-/-}$ thymus (Figure 2) were probably not CD5$^+$IgD$^{-ve}$thymic B cells but macrophage-lineage cells. Perivascular lymphocytic infiltrates in the liver and lung of mice lacking LTα have been described previously (Banks et al., 1995). Thus, the TNF/LTα$^{-/-}$ thymuses were examined histologically to determine whether the increased thymic B cells were present as perivascular infiltrates. This was tested by double staining thymuses for blood vessel-associated connective tissue using MTS 16, which clearly identifies the outer border of perivascular spaces, and B cells using anti-B220. The increased frequency of B cells had no association with vasculature, but rather were localized in the thymic parenchyma (Figure 3 B and C).

### Identification of the Basis of the Increased B Cell Numbers in TNF/LTα$^{-/-}$Mice

To determine the cytokine responsible for increased B cell numbers in the thymus of TNF/LTα$^{-/-}$ mice, the proportion of B220$^+$ thymocytes was examined from mice deficient for either TNF (Korner et al., 1997) or LTα alone (Riminton et al., 1998). The proportion of

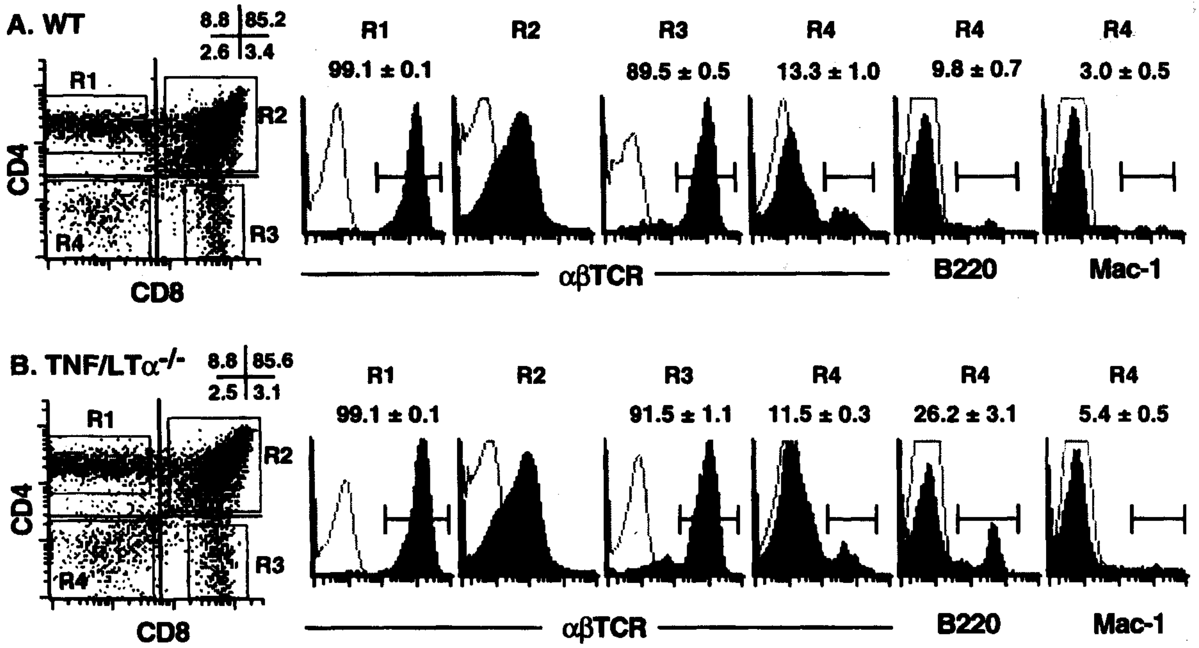

FIGURE 2 Normal T Cell Phenotype but Increased B Cell and Macrophage Incidence in the TNF/LTα$^{-/-}$ Thymus. Thymocytes from WT or TNF/LTα$^{-/-}$ mice were triple-labeled for CD4, CD8 and a third marker and analysed flow cytometrically. Filled histograms indicate αβTCR, B220 and CD11b (Mac-1) staining of the four populations defined by CD4/CD8 labeling (regions (R) 1–4). The unfilled histograms indicate staining of total thymocytes labeled with control hamster mAb (for αβTCR) or isotype-matched control rat mAb (for B220 and CD11b). Flow cytometric data shown is from one WT and one TNF/LTα$^{-/-}$mouse. The proportion of positive cells within each region is given as the mean percent ± SEM of analyses from between 3 and 8 individual mice where n = 8 (αβTCR), n = 6 (B220) and n = 3 (CD11b)

$CD4^-CD8^-B220^+$ cells in TNF$^{-/-}$ thymuses was similar to that of WT thymuses whereas B220$^+$ cells in LTα$^{-/-}$ thymuses were increased (Figure 4) and similar to that observed in the TNF/LTα$^{-/-}$ thymus (Figure 2). This analysis indicated that the lack of LTα rather than TNF was responsible for increased B cell numbers in the TNF/LTα$^{-/-}$ thymus.

To determine whether the increased B cell numbers in the LTα$^{-/-}$ thymus was due to the bone marrow-derived lymphoid component, or to some defect in the thymic stromal elements in the absence of LTα, irradiation, bone marrow chimeras were established where either LTα$^{-/-}$ or WT bone marrow cells were injected into irradiated RAG-1$^{-/-}$ recipient mice and left to enable reconstitution to occur (Figure 4). Despite the presence of WT thymic stroma in both cases, recipients of LTα$^{-/-}$ bone marrow showed increased B cell numbers indicating that defects in the LTα$^{-/-}$ hematopoietic compartment underlay the deposition of increased B cells in the thymus.

### The Influence of TNF/LTα on Negative Selection in vivo

The influence of TNF/LTα on intrathymic negative selection was tested by intrathymic injection of the superantigen SEB. SEB directly binds to members of the Vβ8 TCR family on T cells and MHC-II molecules on antigen presenting cells (Woodland and Blackman, 1993). Intrathymic injection was used because systemic treatment with SEB can lead to non-specific deletion of thymocytes (Lin et al., 1992), possibly due to the increased levels of TNF that follows activation of peripheral T cells. Similar, TNF-mediated, non-specific deletion of non-TCR transgenic DP thymocytes has been reported following peptide-specific stimulation of i.v. adoptively transferred TCR transgenic T cells (Martin and Bevan, 1997). Thus, intrathymic injection of SEB avoided the artefacts associated with systemic T cell stimulation and the generation of levels of TNF sufficient in themselves to cause effects on thymus that would be present in WT but reduced in TNF/LTα$^{-/-}$ mice. The dose of SEB (5μg) was shown in titration studies to effectively delete Vβ8$^{+}$ cells in WT mice when administered into the thymus, whereas the same dose administered i.v. did not (not shown), indicating a local intrathymic effect. Moreover, the proportion of Vβ8$^{+}$ splenic T cells was not affected following intrathymic or i.v. injection of 5μg SEB (M.J. Gabor, manuscript in preparation). The thymus was removed 20 hours following injection and thymocyte subsets defined by CD4 and CD8 expression analysed for the proportion of Vβ8 expressing cells (Figure 5). The extent of Vβ8$^{+}$ thymocyte deletion within CD4$^{+}$CD8$^{-}$ and CD4$^{-}$CD8$^{+}$ subsets in TNF/LTα$^{-/-}$ mice following SEB injection was identical to that of WT mice. SEB treatment did not lead to a decrease in non-SEB reactive Vβ5$^{+}$ CD4 or CD8 SP thymocytes in either WT or TNF/LTα$^{-/-}$ mice, their proportion increasing marginally due to a marked drop in the number of Vβ8$^{+}$ thymocytes.

### DISCUSSION

Several studies have suggested a role for TNF and LT in thymocyte proliferation and differentiation, and in negative selection (Suda et al., 1990; Suda et al., 1990; Suda and Zlotnik, 1992; Suda and Zlotnik, 1992; Zuniga-Pflucker et al., 1995). However, studies using a neutralising anti-TNF antibody, TNF-RI/RII deficient mice, and transgenic mice expressing soluble LTβR or TNFRI have failed to support a role for these factors in thymus physiology (Sytwu et al., 1996; Ettinger et al., 1998; Page et al., 1998). It is important to point out that despite these studies, a detailed analysis of thymic structure and T cell development, including intrathymic positive and negative selection, has not been carried out in the absence of both TNF and LT, hence their potential influence remains unclear. In this study, we have thoroughly examined many aspects of thymic structure, thymocyte differentiation, positive and negative selection in mice rendered genetically deficient for TNF and LTα, The thymus appeared grossly normal, with no difference in cell yield or thymic weight, and no difference in thymic architecture as defined by a range of antibodies against thymocytes and stromal cells. However, we observed consistently a minor albeit significant decrease in cell viability among thymocytes from TNF/LTα$^{-/-}$ mice. It is difficult to be certain what this means, although it is possible that there is reduced macrophage activation due to the absence of TNF, which would slow down the clearance of dead or dying thymocytes that constantly takes place during T cell development (Surh and Sprent, 1994). The absence of any gross structural abnormalities in these thymuses contrasts with that of the spleen and lymph nodes of these mice, where as previously reported, the spleens are devoid of B cell follicles and the lymph nodes are non-existent (Banks et al., 1995; Eugster et al., 1996; Korner et al., 1997), an effect substantially due to the absence of LT (De Togni et al., 1994; Banks et al., 1995; Ettinger et al., 1996; Mackay et al., 1997). Thus, factors regulating the generation and/or maintenance of the thymus are clearly distinct from those controlling the peripheral lymphoid organs.

Most thymocyte subsets were present in normal proportions. This suggests that the transition through early CD3/αβTCR$^{-}$CD4$^{-}$CD8$^{-}$triple negative (TN) stages, and from the TN to the DP stage does not absolutely depend on either TNF or LTα, despite earlier studies in cell suspension culture showing a positive influence for TNF on these immature cells (Suda et al., 1990; Suda et al., 1990; Suda and Zlotnik, 1992; Suda and Zlotnik, 1992; Zuniga-Pflucker et al., 1995). It is possible that the functions of TNF/LTα are redundant and replaced by other factors in these mice, but it should also be considered that the influence of TNF in the earlier studies (all based upon in vitro experiments) does not represent a physiological role for this factor at this stage of T cell development. It should be pointed out that although the total CD4$^{-}$CD8$^{-}$ population was normal in TNF/LTα$^{-/-}$ mice, the increased proportion of B cells and macrophages within this compartment must mean that some other CD4$^{-}$CD8$^{-}$ cells are correspondingly diminished. Although a small decrease was detected in the

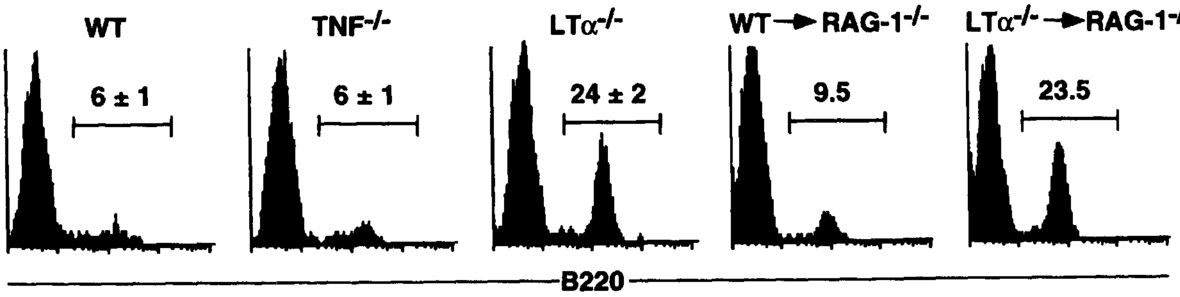

FIGURE 4 Increased Thymic B Cells are due to LTα Deficiency in the Lymphoid Compartment of the Thymus. Thymocytes from mice as indicated were triple-labelled for CD4, CD8 and B220 expression. Histogram profiles represent B220 expression on $CD4^-CD8^-$ thymocytes from mice as indicated. The fourth and fifth histograms represent thymocytes from irradiated C57BL/6 $RAG\text{-}1^{-/-}$ mice that were were reconstituted with bone marrow cells derived from either WT or $LT\alpha^{-/-}$ C57BL/6 mice. Reconstituted thymuses were assessed 12 weeks later, when engraftment was complete. Histogram profiles of WT, $TNF^{-/-}$ and $LT\alpha^{-/-}$ thymocytes are representative of at least 3 different mice. The fourth histogram is derived from 1 mouse and the fifth representative of 2 mice frequency of $\alpha\beta TCR^+CD4^-CD8^-$ cells, this is unlikely to account for the difference. As lineage marker (Mac-1, B220, Gr-1, TER119, CD3, CD4, CD8) negative cells, representing real thymocyte precursors (Godfrey and Zlotnik, 1993) were not directly investigated it is possible that these cells were also reduced. However, if this was the case it had no apparent downstream effects on subsequent T cell subsets.

The increased presence of B cells in the thymus due to LTα deficiency was an unexpected result. This may be due to dysregulated control over the point at which thymocytes lose their multilineage potential, believed to occur between the $CD44^+CD25^-$ and $CD44^+CD25^+$ TN stages (Godfrey and Zlotnik, 1993), leading to an increase in B cell differentiation from early thymocyte precursors. However, thymic B cells are normally considered to be B1 type cells ($CD5^+$), whereas the increased B cells detected in the present study more closely resembled conventional B2 type cells, as found in spleen and lymph nodes (Miyama-Inaba et al., 1988). An alternative possibility therefore is that LTα is important in controlling the trafficking or proliferation of peripheral B cells that have found their way to the thymus. A more mundane explanation is that the three-fold increase in circulating leukocytes seen in $LT\alpha^{-/-}$ mice (Banks et al., 1995; Riminton et al., 1998) may simply lead to a spill-over of B cells (and monocytes, Figure 2) into the thymus. Perivascular accumulations of lymphocytes are seen in some tissues in $LT\alpha^{-/-}$ mice (Banks et al., 1995). Notably however, the B cells were not localized to the perivascular space, but deeper within the tissue (Figure 3). In the absence of a clearer understanding of the ontogeny and regulation of B cells in the thymus, it is difficult to speculate any further on the basis of the B cell increase in $LT\alpha^{-/-}$ mice.

Our studies have also shown that positive selection is not associated with TNF/LTα signalling, as post selection $CD4^+CD8^-$ and $CD4^-CD8^+$ thymocytes were present in normal proportions. This is not a contentious issue, as these factors have never been associated with positive selection. A more equivocal problem is the role of TNF and LT in intrathymic negative selection, with evidence for (Hernandez-Caselles and Stutman, 1993; Page et al., 1996; Page et al., 1998) and against (Sytwu et al., 1996; Page et al., 1998) a role for TNF in this process, and no direct evidence either way for LT. The results presented in this manuscript are the most definitive to date, as negative selection has been studied in an *in vivo* thymic microenvironment which is structurally normal, yet completely deficient in both TNF and membrane LTαβ or secreted LTα3. These results show that negative selection induced by the superantigen SEB, was clearly TCR-mediated and completely

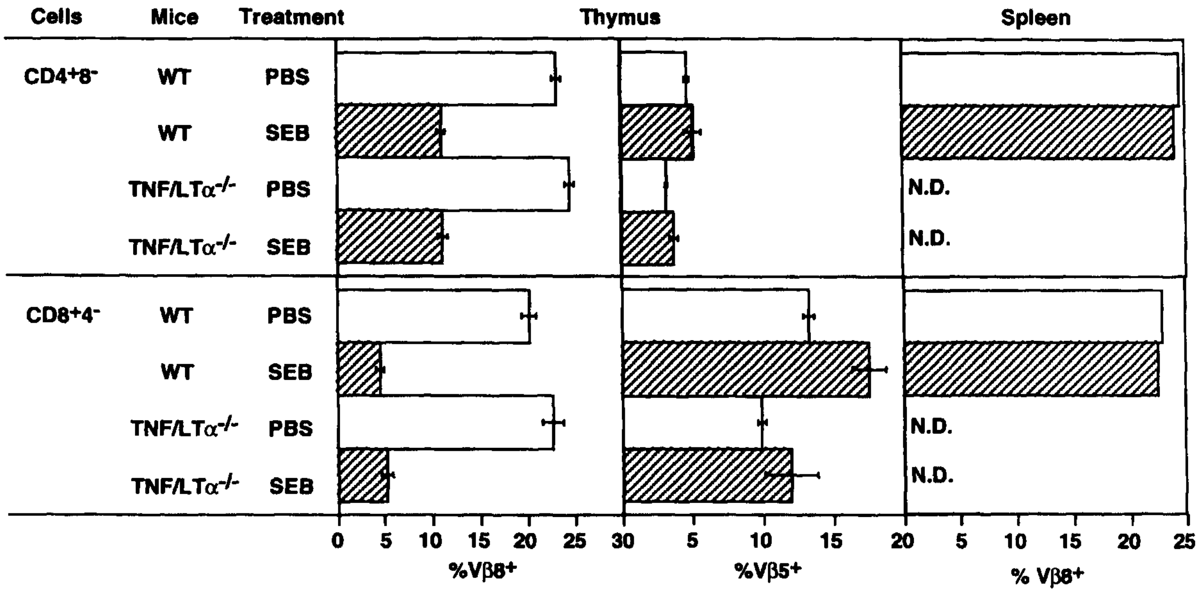

FIGURE 5 Intrathymic SEB Induces Specific T Cell Deletion in TNF/LTα$^{-/-}$ Mice. Groups of three WT and TNF/LTα$^{-/-}$ mice were anesthetized, the thymus exposed surgically and injected with either PBS or 5μg SEB in PBS (10μl), then the incision closed with a single surgical staple. Twenty hours later, mice were killed and tissues obtained for analysis. Cells were triple-labeled for CD4, CD8 and either Vβ8 (mAb F23.1; 8.1, 8.2, 8.3) or Vβ5 (mAb MR9–4) and analysed by flow cytometry. Bars represent the percentage (mean ± 1 SEM, where appropriate) of Vβ8$^+$ or Vβ5$^+$ cells within the CD4$^+$CD8$^-$ or CD8$^+$CD4$^-$ thymocyte populations. The data shown here represents the outcome of a single experiment in which all mice were injected with the same SEB preparation on the same day and then all mice sacrificed and cell phenotype examined together on the following day. A second study in which groups of two mice were examined produced a qualitatively identical outcome but with a marginally reduced magnitude of SEB-induced deletion (PBS-injected thymus essentially as per this figure. SEB-injected thymus, CD4$^+$CD8$^-$Vβ8$^+$ = mean of 13%. CD4$^-$CD8$^+$Vβ8$^+$ = mean of 7%). Representative spleen data derived from a single experiment is shown. N.D. = not done independent of these factors. Although superantigens such as SEB do not bind to the same part of the T cell receptor as conventional antigen (Woodland and Blackman, 1993), they do require costimulatory signals such as CD28 for the activation of peripheral T cells, suggesting a similar interaction between developing thymocytes and thymic antigen presenting cells involved in deletion. Clearly, it is technically very difficult to measure negative selection in response to a conventional antigen in non-TCR transgenic mice, and TCR transgenic TNF/LTα$^{-/-}$ C57BL/6 mice were not available. Furthermore, analysis of negative selection in such mice would require at least two mouse lines including an MHC class I and MHC class II-restricted TCR transgene to study selection of both CD4 and CD8 T cells.

Our results clearly demonstrated the rapid deletion of many SEB-reactive thymocytes within 20 hours of exposure to this superantigen. This included both less mature DP thymocytes (not shown) but was more apparent in the CD4 and CD8 SP thymocyte subsets, due to the significantly higher levels of TCR expression in this compartment. Although early studies had suggested that most negative selection occurs at the DP stage rather than the SP stage of T cell development (Kisielow and von Boehmer, 1990), this is clearly not absolute (Surh and Sprent, 1994; Kishimoto and Sprent, 1997; Kishimoto et al., 1998). The reason why some SP thymocytes survived this deletion following SEB encounter is uncertain, although it may reflect variations in the level of maturity among these cells, such that the most mature ones are resistant to deletion, as previously reported following anti-CD3 and SEB challenge *in vivo* (Kishimoto and Sprent, 1997; Kishimoto et al., 1998).

Taken together, this study provides the most definitive results to date of the role of TNF and LT in thymic function, clearly showing that both cytokines are dispensable for most aspects of intrathymic T cell differentiation, including early thymocyte differentiation, positive and negative selection and maintenance of thymic structure. However, the results indicate minor roles for these factors, including maintenance of cell viability in the thymus, and possibly a role for LTα in the generation or maintenance of B cell numbers in the thymus.

### MATERIALS AND METHODS

### Animals

Specific pathogen free WT female C57BL/6 and C57BL/6 RAG-1$^{-/-}$ (Spanopoulou et al., 1994) mice were purchased from the Animal Resources Centre, Perth, Australia. C57BL/6-strain TNF$^{-/-}$, LTα$^{-/-}$, and TNF/LTα$^{-/-}$ mice were generated by direct targeting of C57BL/6 embryonic stem cells at the Centenary Institute, Sydney, Australia as described (Korner et al., 1997; Riminton et al., 1998). Mice were housed under specific pathogen free conditions in the Centenary Institute Animal Facility and used at 6–10 weeks of age.

### Cell Suspensions

Tissues were removed and gently ground between the frosted ends of microscope slides in PBS containing 5% FCS and 0.02% Na-Azide. Cells were washed by pelleting and resuspending in the same buffer. Cell numbers and viability were determined using a hemocytometer and Trypan blue (Sigma Chemical Co. Castle Hill, NSW, Australia) dye exclusion.

### Flow Cytometry

Lymphocyte cell suspensions were stained with fluorescent antibodies to mouse markers: anti-CD4-phycoerythrin (PE) or allophycocyanin (clone RM4–5; Pharmingen, San Diego, CA. USA); anti-CD4-FITC or tricolor (clone CT-CD4; Caltag Laboratories, Burlingame, CA. USA); anti CD8-FITC or tricolor (clone CT-CD8; Caltag Laboratories); anti-αβTCR-FITC or PE (clone H57–597; Pharmingen); anti-γδTCR-FITC (clone GL3; Pharmingen); anti-HSA-PE (clone M1/69; Pharmingen); anti NK1.1-PE (clone PK136; Pharmingen); anti Mac-1-PE (clone M1/70; Pharmingen); anti B220-PE (clone RA3–6B2; Caltag Laboratories); anti-CD5-biotin (clone 53–7.8; a gift of Dr. Paul Lalor, Walter and Eliza Hall Institute, Melbourne, Australia, grown and conjugated in house at Centenary Institute) detected using streptavidin-FITC (Pharmingen); anti-CD11c (clone N4.18; grown in house at Centenary Institute, courtesy of Dr. Deborah Strickland) detected using goat anti-hamster Ig-FITC (Caltag Laboratories); anti-IgD (clone AF6–122.2; courtesy of Dr. Paul Lalor, grown in house at Centenary Institute), detected using goat anti-rat Ig-FITC (Caltag); anti-Vβ8 (clone F23.1; grown in house at Centenary Institute) detected using sheep anti-mouse Ig-FITC (Sigma Chemical Co); anti-Vβ5-FITC (clone MR9–4). Isotype controls included rat IgG2b-FITC (clone R35–95; Pharmingen); rat IgG2a (clone R35–38; Pharmingen); polyclonal hamster Ig-FITC (Caltag Laboratories). Fluorescence data was obtained using a FACScan™ (Becton Dickinson, San Jose CA. USA) or FACStar Plus™ (Becton Dickinson) and analysed using CELLQuest 3.0 software™ (Becton Dickinson).

### Immunohistochemistry

Six μm cryosections were labelled with unconjugated rat anti-mouse antibodies including anti-CD4 (clone GK1.5); anti-CD8 (clone 53–6.7); anti-Mac-1 (clone M1/70); anti-B220 (clone 5H-3, grown in-house at Centenary Institute); anti MHC class II (I-A$^b$) (clone Tib 120); anti-thymic stromal cell markers MTS 12, MTS 16, MTS 35 (courtesy of Dr. Richard Boyd, Monash University). These unconjugated antibodies were detected using rabbit anti-rat-Ig-horseradish peroxidase (HRP) (DAKO, Australia). Other antibodies used included: hamster anti-mouse CD11c (clone

N4.18) detected using goat anti-hamster Ig-biotin; and anti-B220-biotin (5H-3, Centenary Institute). Biotinylated antibodies were detected using streptavidin-HRP or streptavidin alkaline phosphatase (AP) (DAKO). Negative control antibodies included rat IgG2a (clone R35–95; Pharmingen), rat IgG2b (clone YKIX) (grown in house at Centenary Institute), and hamster IgG (clone UC8–4b3; Pharmingen). Single and two color immunohistological procedures were as described (Sedgwick et al., 1993).

### Intrathymic Injections with SEB

Mice were anesthetized by i.p. injection of (0.75mg) ketamine hydrochloride (Ketapex, Apex Laboratories Pty. Ltd. St. Marys, NSW, Australia) and (0.35 mg) Xylazine (Rompun, Bayer Ltd. Pymble, NSW. Australia). Once anesthetized, the thoracic cavity was opened via a small midline incision, and 5 μg of SEB (Sigma) in 10 μl of PBS, or PBS alone, was injected intrathymically. The incision was closed using a single surgical staple and the mice injected with 0.02 mg Buprenorphine analgesic (Temgesic, Reckitt & Colman Products Ltd. Hull, UK) and allowed to recover in a warm environment. Mice were killed 20 hours after treatment and tissues harvested.

### Generation of Radiation Bone Marrow Chimeras

C57BL/6 RAG-$1^{-/-}$ mice were lethally irradiated (550 rad of γ-radiation day −2 and day 0) and on day 0, injected i.v. with $2 \times 10^7$ bone marrow cells derived from either WT or $LT\alpha^{-/-}$ C57BL/6 mice. Thymuses of mice were assessed 12 weeks later, when engraftment was complete (Riminton et al., 1998).

### *Acknowledgements*

The authors thank Dr Heinrich Körner (Centenary Institute, Sydney) for generation and maintenance of the $TNF^{-/-}$ and $LT\alpha^{-/-}$ mouse lines and for helpful discussions throughout the course of this work, Dr Roland Scollay for his support in the early phases of these studies, Dr Richard Boyd and Mr Mark Malin for provision of the MTS mAb. Thanks are also due to Dr. Alan Baxter and Mr. Stuart Berzins for helpful discussions during the preparation of this manuscript. The outstanding management of the Centenary Institute Animal Facility and direct animal care by Ms Karen Knight and Mr James Croser respectively, is acknowledged. These studies were supported by a Program Grant (#953216 to JDS), and Project Grant (#970603 to DIG) from the National Health and Medical Research Council (NHMRC), a Project Grant to JDS from the National Multiple Sclerosis Society of Australia, an R.D. Wright Fellowship to DIG from the NHMRC, and by post-graduate studentships to MJG (Australian Post-Graduate Award) and DSR (NHMRC Post-Graduate Research Award).

### *References*

Banks T.A., Rouse B.T., Kerley M.K., Blair P.J., Godfrey V.L., Kuklin N.A., Bouley D.M., Thomas J., Kanangat S. and Mucenski M.L. (1995). Lymphotoxin-alpha-deficient mice – effects on secondary lymphoid organ development and humoral immune responsiveness. J. Immunol. **155**:1685–1693.

Browning J.L., Miatkowski K., Griffiths D.A., Bourdon P.R., Hession C., Ambrose C.M. and Meier W. (1996). Preparation and characterization of soluble recombinant heterotrimeric complexes of human lymphotoxins alpha and beta. J. Biol. Chem. **271**:8618–8626.

Browning J.L., Ngam-ek A., Lawton P., DeMarinis J., Tizard R., Chow E.P., Hession C., O'Brine-Greco B., Foley S.F. and Ware C.F. (1993). Lymphotoxin beta, a novel member of the TNF family that forms a heteromeric complex with lymphotoxin on the cell surface. Cell **72**:847–856.

Cook M., Körner H., Riminton D.S., Lemckert F.A., Hasbold J., Amesbury M., Hodgkin P.D., Cyster J.G., Sedgwick J.D. and Basten A. (1998). Generation of splenic follicular structure and B cell movement in tumor necrosis factor-deficient mice. J. Exp. Med. **188**:1503–1510.

De Togni P., Goellner J., Ruddle N.H., Streeter P.R., Fick A., Mariathasan S., Smith S.C., Carlson R., Shornick L.P., Strauss-Schoenberger J., Russel J.H., Karr R. and Chaplin D.D. (1994). Abnormal development of peripheral lymphoid organs in mice deficient in lymphotoxin. Science **264**:703–707.

Deman J., Van Meurs M., Claassen E., Humblet C., Boniver J. and Defresne M.P. (1996). In vivo expression of interleukin-1 beta (IL-1 beta), IL-2, IL-4, IL-6, tumour necrosis factor-alpha and interferon-gamma in the fetal murine thymus. Immunology **89**:152–157.

Ettinger R., Browning J.L., Michie S.A., VanEwijk W. and McDevitt H.O. (1996). Disrupted splenic architecture, but normal lymph node development in mice expressing a soluble lymphotoxin-beta receptor-iggl fusion protein. Proc. Natl. Acad. Sci. USA **93**:13102–13107.

Ettinger R., Mebius R., Browning J.L., Michie S.A., VanTuijl S., Kraal G., VanEwijk W. and McDevitt H.O. (1998). Effects of tumor necrosis factor and lymphotoxin on peripheral lymphoid tissue development. Int. Immunol. **10**:727–741.

Eugster H.P., Muller M., Karrer U., Car B.D., Schnyder B., Eng V.M., Woerly G., Le Hir M., di Padova F., Aguet M., Zinkernagel R., Bluethmann H. and Ryffel B. (1996). Multiple immune abnormalities in tumor necrosis factor and lymphotoxin-alpha double-deficient mice. Int. Immunol. **8**:23–36.

Fischer M., MacNeil I., Suda T., Cupp J.E., Shortman K. and Zlotnik A. (1991). Cytokine production by mature and immature thymocytes. J Immunol **146**:3452–3456.

Gabor M.J., Scollay R. and Godfrey D.I. (1997). Thymic T cell export is not influenced by the peripheral T cell pool. Eur. J. Immunol. **27**:2986–2993.

Giroir B.P., Brown T. and Beutler B. (1992). Constitutive synthesis of tumor necrosis factor in the thymus. Proc. Natl. Acad. Sci. USA **89**:4864–4868.

Godfrey D.I., Izon D.J., Tucek C.L., Wilson T.J. and Boyd R.L. (1990). The phenotypic heterogeneity of mouse thymic stromal cells. Immunology **70**:66–74.

Godfrey D.I. and Zlotnik A. (1993). Control points in early T-cell development. Immunol Today **14**:547–553.

Grell M., Becke F.M., Wajant H., Mannel D.N. and Scheurich P. (1998). TNF receptor type 2 mediates thymocyte proliferation independently of TNF receptor type 1. Eur. J. Immunol. **28**:257–263.

Hardy R.R. and Hayakawa K. (1994). CD5 B cells, a fetal B cell lineage. Adv. Immunol. **55**:297–339.

Hernandez-Caselles T. and Stutman O. (1993). Immune functions of tumor necrosis factor. I. Tumor necrosis factor induces apoptosis of mouse thymocytes and can also stimulate or inhibit IL-6-induced proliferation depending on the concentration of mitogenic costimulation. J. Immunol. **151**:3999–4012.

Kipps T.J. (1989). The CD5 B cell. Adv. Immunol. **47**:117–185.

Kishimoto H. and Sprent J. (1997). Negative selection in the thymus includes semimature T cells. J. Exp. Med. **185**:263–271.

Kishimoto H., Surh C.D. and Sprent J. (1998). A role for FAS in negative selection of thymocytes in vivo. J. Exp. Med. **187**:1427–1438.

Kisielow P. and von Boehmer H. (1990). Negative and positive selection of immature thymocytes: timing and the role of the ligand for alpha beta T cell receptor. Sem. Immunol. **2**:35–44.

Kisielow P. and von Boehmer H. (1995). Development and selection of T cells: facts and puzzles. Adv. Immunol. **58**:87–210.

Koni P.A., Sacca R., Lawton P., Browning J.L., Ruddle N.H. and Flavell R.A. (1997). Distinct roles in lymphoid organogenesis for lymphotoxins alpha and beta revealed in lymphotoxin beta-deficient mice. Immunity **6**:491–500.

Korner H., Cook M., Riminton S., Lemckert F.A., Hoek R.M., Lederman B., Kontgen F., Fazekas de St Groth B. and Sedgwick J.D. (1997). Distinct role for lymphotoxin alpha and tumor necrosis factor in organogenesis and spatial organisation of lymphoid tissue. Eur. J. Immunol. **27**:2600–2609.

Levitsky H.I., Golumbek P.T. and Pardoll D.M. (1991). The fate of CD4-8- T cell receptor-αβ+ thymocytes. J. Immunol. **146**:1113–1117.

Lin Y.S., Lei H.Y., Low T.L., Shen C.L., Chou L.J. and Jan M.S. (1992). In vivo induction of apoptosis in immature thymocytes by staphylococcal enterotoxin B. J Immunol **149**:1156–1163.

Mackay F., Majeau G.R., Lawton P., Hochman P.S. and Browning J.L. (1997). Lymphotoxin but not tumor necrosis factor functions to maintain splenic architecture and humoral responsiveness in adult mice. Eur. J. Immunol. **27**:2033–2042.

Martin S. and Bevan M.J. (1997). Antigen specific and non-specific deletion of immature cortical thymocytes caused by antigen injection. Eur. J. Immunol. **27**:2726–2736.

Miyama-Inaba M., Kuma S., Inaba K., Ogata H., Iwai H., Yasumizu R., Muramatsu S., Steinman R.M. and Ikehara S. (1988). Unusual phenotype of B cells in the thymus of normal mice. J. Exp. Med. **168**:811–816.

Mori S., Inaba M., Sugihara A., Taketani S., Doi H., Fukuba Y., Yamamoto Y., Adachi Y., Inaba K., Fukuhara S. and Ikehara S. (1997). Presence of B cell progenitors in the thymus. J. Immunol. **158**:4193–4199.

Murphy M., Friend D.S., Pike Nobile L. and Epstein L.B. (1992). Tumor necrosis factor-alpha and IFN-gamma expression in human thymus. Localization and overexpression in Down syndrome (trisomy 21). J. Immunol. **149**:2506–2512.

Murphy M., Pikenobile L., Soo V.W. and Epstein L.B. (1994). Characterization of tnf receptors on human thymocytes. Thymus **23**:177–194.

Nagata S. (1997). Apoptosis by death factor. Cell **88**:355–365.

Ngo V.N., Körner H., Gunn M.D., Schmidt K.N., Riminton D.S., Cooper M.D., Browning J.L., Sedgwick J.D. and Cyster J.G. (1999). Lymphotoxin-αβ and tumor necrosis factor are required for stromal cell expression of homing chemokines in B and T cell areas of the spleen. J. Exp. Med. **189**:403–412.

Page D.M., Kane L.P., Allison J.P. and Hedrick S.M. (1993). Two signals are required for negative selection of CD4+CD8+ thymocytes. J. Immunol. **151**:1868.

Page D.M., Kane L.P., Onami T.M. and Hedrick S.M. (1996). Cellular and biochemical requirements for thymocyte negative selection. Sem. Immunol. **8**:69–82.

Page D.M., Roberts E.M., Peschon J.J. and Hedrick S.M. (1998). TNF receptor deficient mice reveal striking differences between several models of thymocyte negative selection. J. Immunol. **160**:120–133.

Pasparakis M., Alexopoulou L., Episkopou V. and Kollias G. (1996). Immune and inflammatory responses in TNF alpha-deficient mice: a critical requirement for TNF alpha in the formation of primary B cell follicles, follicular dendritic cell networks and germinal centers, and in the maturation of the humoral immune response. J. Exp. Med. **184**:1397–1411.

Pfeffer K., Matsuyama T., Kundig T.M., Wakeham A., Kishihara K., Shahinian A., Wiegmann K., Ohashi P.S., Kronke M. and Mak T.W. (1993). Mice deficient for the 55kD tumour necrosis factor receptor are resistant to endotoxic shock, yet succumb to L. monocytogenes infection. Cell **73**:457–467.

Pokholok D.K., Maroulakou I.G., Kuprash D.V., Alimzhanov M.B., Kozlov S.V., Novobrantseva T.I., Turetskaya R.L., Green J.E. and Nedospasov S.A. (1995). Cloning and expression analysis of the murine lymphotoxin beta gene. Proc. Natl. Acad. Sci. USA **92**:674–678.

Probert L., Keffer J., Corbella P., Cazlaris H., Patsavoudi E., Stephens S., Kaslaris E., Kioussis D. and Kollias G. (1993). Wasting, ischemia, and lymphoid abnormalities in mice expressing T cell targeted human tumor necrosis factor transgenes. J. Immunol. **151**:4637.

Raviola E. and Karnovsky M.J. (1972). Evidence for a blood thymus barrier using electron opaque tracers. J. Exp. Med. **136**:466–473.

Riminton D.S., Korner H., Strickland D.H., Lemckert F.A., Pollard J.D. and Sedgwick J.D. (1998). Challenging cytokine redundancy: Inflammatory cell movement and clinical course of experimental autoimmune encepahlomyelitis are normal in lymphotoxin-deficient but not TNF-deficient mice. J. Exp. Med. **187**:1517–1528.

Ryffel B., Brockhaus M., Greiner B., Mihatsch M.J. and Gudat F. (1991). Tumour necrosis factor receptor distribution in human lymphoid tissue. Immunology **74**:446–452.

Sarin A., Cona-Cibotti M. and Henkart P.A. (1995). Cytotoxic effect of TNF and lymphotoxin on T lymphoblasts. J. Immunol. **155**:3716–3718.

Sedgwick J.D., Schwender S., Gregersen R., Dörries R. and ter Meulen V. (1993). Resident macrophages (ramified microglia) of the BN-strain rat central nervous system are constitutively MHC class II-positive. J. Exp. Med. **177**:1145–1152.

Spanopoulou E., Roman C.A., Corcoran L.M., Schlissel M.S., Silver D.P., Nemazee D., Nussenzweig M.C., Shinton S.A., Hardy R.R. and Baltimore D. (1994). Functional immunoglobulin transgenes guide ordered B-cell differentiation in Rag-1-deficient mice. Genes Develop. **8**:1030–1042.

Suda T., Murray R., Fischer M., Yokota T. and Zlotnik A. (1990). Tumor necrosis factor-alpha and P40 induce day 15 murine fetal thymocyte proliferation in combination with IL-2. J Immunol **144**:1783–1787.

Suda T., Murray R., Guidos C. and Zlotnik A. (1990). Growth-promoting activity of IL-1 alpha, IL-6, and tumor necrosis factor-alpha in combination with IL-2, IL-4, or IL-7 on murine thymocytes. Differential effects on CD4/CD8 subsets and on CD3+/CD3- double-negative thymocytes. J Immunol **144**:3039–3045.

Suda T. and Zlotnik A. (1992). In vitro induction of CD8 expression on thymic pre-T cells. I. Transforming growth factor-beta and tumor necrosis factor-alpha induce CD8 expression on CD8- thymic subsets including the CD25+CD3-CD4-CD8- pre-T cell subset. J Immunol **148**:1737–1745.

Suda T. and Zlotnik A. (1992). In vitro induction of CD8 expression on thymic pre-T cells. II. Characterization of CD3-CD4-CD8 alpha + cells generated in vitro by culturing CD25+CD3-CD4-CD8- thymocytes with T cell growth factor-beta and tumor necrosis factor-alpha. J Immunol **149**:71–76.

Surh C.D. and Sprent J. (1994). T-cell apoptosis detected in situ during positive and negative selection in the thymus. Nature **372**:100–103.

Sytwu H.K., Liblau R.S. and McDevitt H.O. (1996). The roles of Fas/APO-1 (CD95) and TNF in antigen-induced programmed cell death in T cell receptor transgenic mice. Immunity **5**:17–30.

Tartaglia L.A., Weber R.F., Figari I.S., Reynolds C., Palladino M.A. and Goeddel D.V. (1991). The two different receptors for tumor necrosis factor mediate distinct cellular responses. Proc. Natl. Acad. Sci. USA **88**:9292–9296.

Ware C.F., VanArsdale T.L., Crowe P.D. and Browning J.L. (1995). The ligands and receptors of the lymphotoxin system. Curr. Top. Microbiol. Immunol. **198**:175–218.

Wolf S.S. and Cohen A. (1992). Expression of cytokines and their receptors by human thymocytes and thymic stromal cells. Immunology **77**:362–368.

Woodland D.L. and Blackman M.A. (1993). How do T-cell receptors, MHC molecules and superantigens get together? Immunol Today **14**:208–212.

Zlotnik A., Godfrey D.I., Fischer M. and Suda T. (1992). Cytokine production by mature and immature CD4-CD8- T cells. Alpha beta-T cell receptor+ CD4-CD8- T cells produce IL-4. J Immunol **149**:1211–1215.

Zuniga-Pflucker J.C., Jiang D. and Lenardo M.J. (1995). Requirement for TNF-alpha and IL-1-alpha in fetal thymocyte commitment and differentiation. Science **268**:1906–1909.

